# Supplementary material for: Genome-wide prediction of bacterial effector candidates across six secretion system types using a feature-based statistical framework
Source: Sci Rep. 2018 Nov 21;8:17209. doi: 10.1038/s41598-018-33874-1 (PMC6249201; doi:10.1038/s41598-018-33874-1)
Supplement: Supplementary file 1 — Supplementary Information [file 41598_2018_33874_MOESM1_ESM.doc]

**SUPPLEMENTARY INFORMATION**

**Genome-wide prediction of bacterial effector candidates across six secretion system types using a feature-based statistical framework**

Andi Dhroso^1^, Samantha Eidson^2^, and Dmitry Korkin^1,*^

^1^Department of Computer Science, and Bioinformatics and Computational Biology Program, Worcester Polytechnic Institute, Worcester, MA, USA

^2^Mathematics and Computer Science Department, Fontbonne University, St. Louis, MO, USA

*To whom correspondence should be addressed.


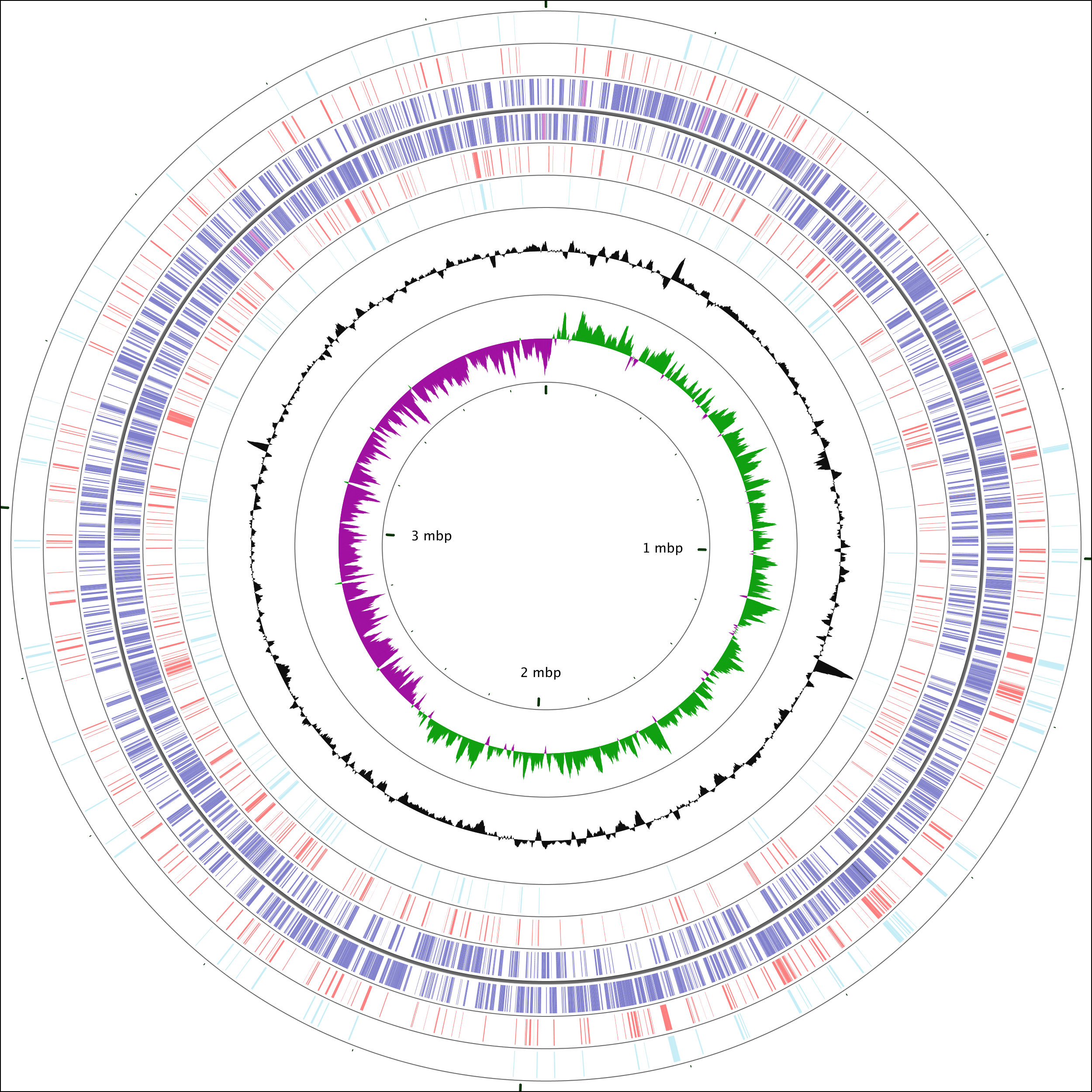


**Figure S1 – Predicted effector candidates of *Acinetobacter baumannii*.** Effector candidates were predicted using the default optimal model (Random Forest, red) and SVM with extremely stringent threshold (*θ*=0.9, cyan) are mapped according to their corresponding positions on the circular bacterial genome. All known genes (blue) are mapped on the corresponding DNA strand of the genome. Shown in black is GC content. Shown in green and purple are GC skew+ and GC skew-, respectively. Regions of tightly clustered effector candidates can be clearly identified. The image was generated using CGView Comparison Tool.


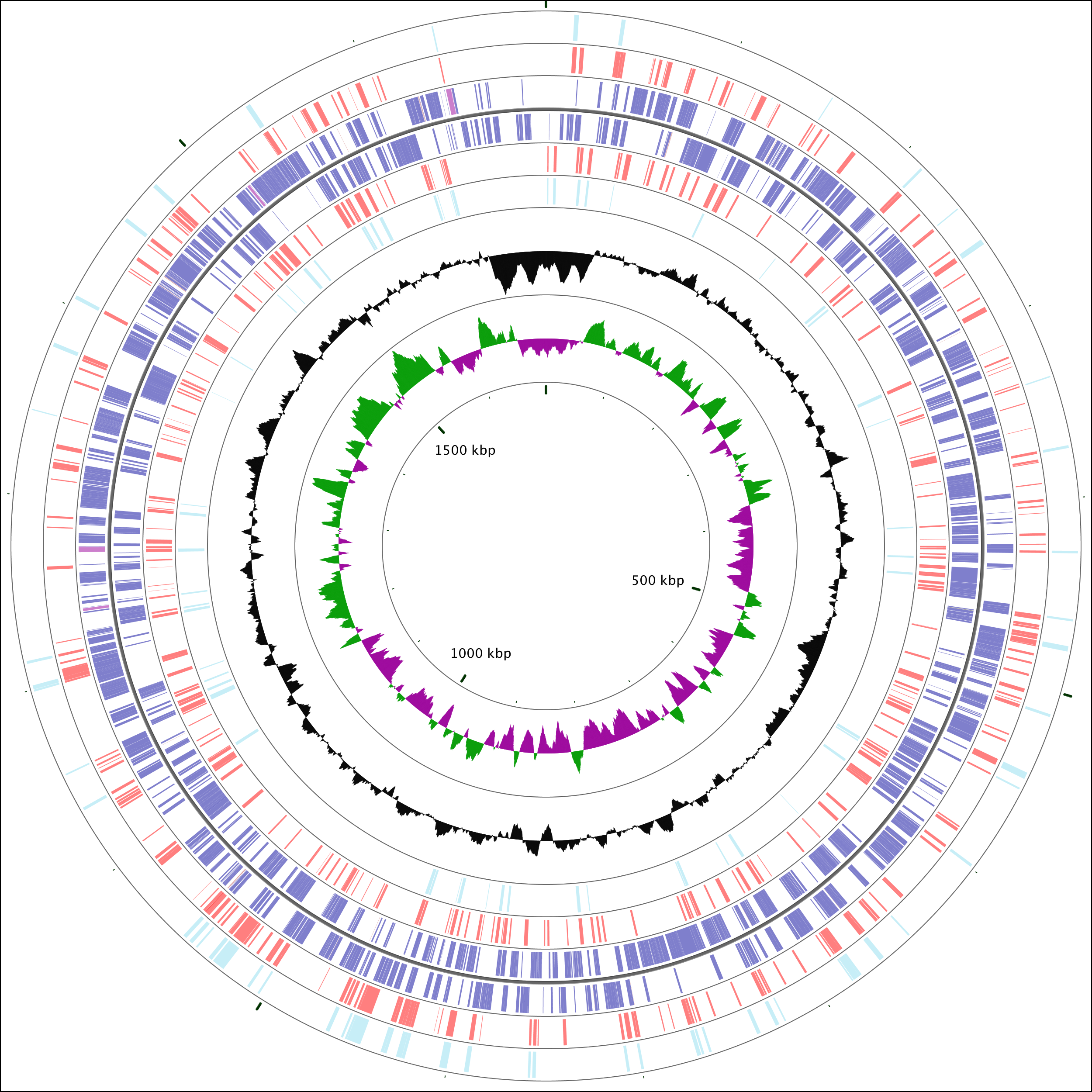


**Figure S2 – Predicted effector candidates** **of *Helicobacter pylori*.** Effector candidates were predicted using the default optimal model (Random Forest, red) and SVM with extremely stringent threshold (*θ*=0.9, cyan) are mapped according to their corresponding positions on the circular bacterial genome. The mapping protocol and color annotation are identical to those ones in Figure S1.


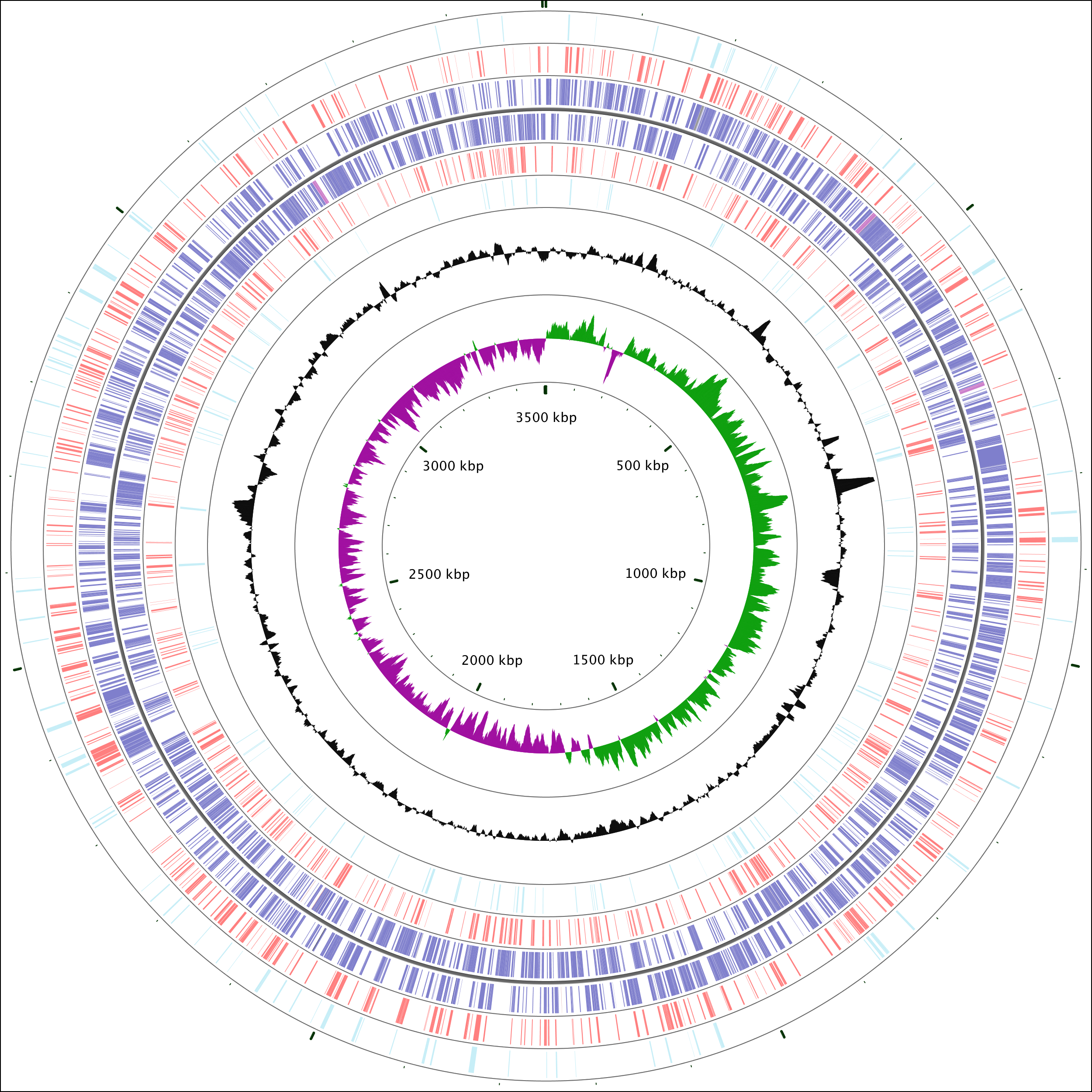


**Figure S3 – Predicted effector candidates of *Legionella pneumophila*.** Effector candidates were predicted using the default optimal model (Random Forest, red) and SVM with extremely stringent threshold (*θ*=0.9, cyan) are mapped according to their corresponding positions on the circular bacterial genome. The mapping protocol and color annotation are identical to those ones in Figure S1.


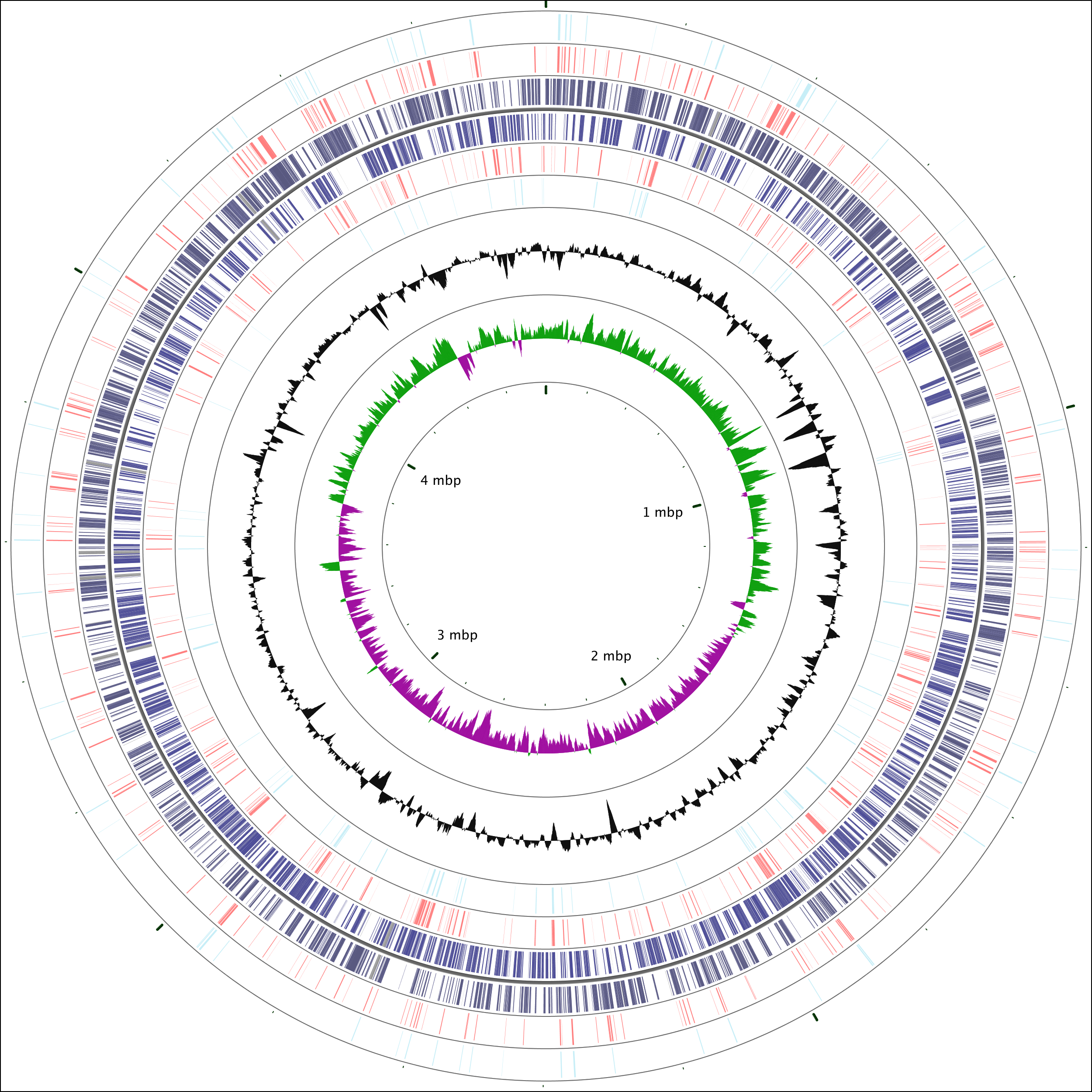


**Figure S4 – Predicted effector candidates of *Salmonella enterica*.** Effector candidates were predicted using the default optimal model (Random Forest, red) and SVM with extremely stringent threshold (*θ*=0.9, cyan) are mapped according to their corresponding positions on the circular bacterial genome. The mapping protocol and color annotation are identical to those ones in Figure S1.


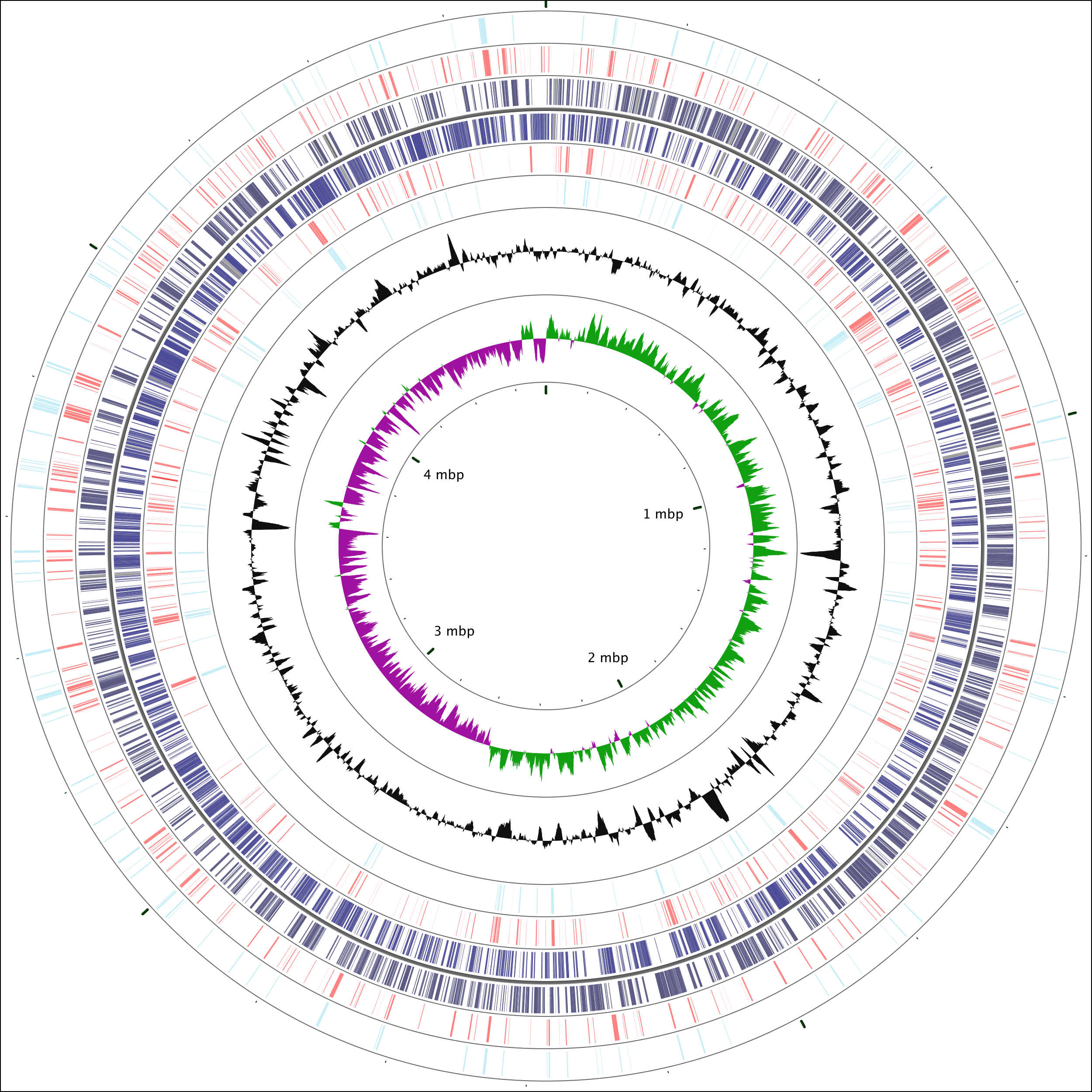


**Figure S5 – Predicted effector candiodates of *Yersinia pseudotuberculosis*.** Effector candidates were predicted using the default optimal model (Random Forest, red) and SVM with extremely stringent threshold (*θ*=0.9, cyan) are mapped according to their corresponding positions on the circular bacterial genome. The mapping protocol and color annotation are identical to those ones in Figure S1.

**Table S1. Distribution of effectors in the positive set across six secretion system types.** To exclude bias, the effectors are evenly distributed across the secretion systems and are selected from different species.

| **Secretion System** | **Positive Set Size** | **N of species** |
| --- | --- | --- |
| I | 20 | 10 |
| II | 30 | 11 |
| III | 31 | 13 |
| IV | 30 | 8 |
| V | 27 | 12 |
| VI | 30 | 17 |
| Total | 168 | 71 |

**Table S2. Distribution of positive (effectors) and negative (non-effectors) sets across different genera.** Reported for each genus are the numbers of effectors, non-effectors, and secretion systems that those effectors belong to.

| **Genus** | **Secretion  System(s)** | **N of effectors** | **N of non-effectors** |
| --- | --- | --- | --- |
| *Acidovorax* | 3 | 1 | 5 |
| *Acinetobacter* | 2 | 2 | 6 |
| *Aeromonas* | 2,3,6 | 12 | 5 |
| *Aggregatibacter* | 5 | 2 | 5 |
| *Agrobacterium* | 4,6 | 6 | n/a |
| *Anaplasma* | 4 | 4 | 5 |
| *Bartonella* | 4 | 7 | 5 |
| *Bordetella* | 1,3,4,5 | 13 | 5 |
| *Burkholderia* | 2,3,6 | 17 | 10 |
| *Caulobacter* | 1,3 | 2 | n/a |
| *Chlamydia* | 3 | 3 | 5 |
| *Coxiella* | 4 | 5 | 5 |
| *Dichelobacter* | 4,5 | 4 | 4 |
| *Dickeya* | 6 | 1 | n/a |
| *Edwardsiella* | 3,6 | 3 | 1 |
| *Ehrlichia* | 1,4 | 5 | 5 |
| *Erwinia* | 1,2,3,6 | 23 | 2 |
| *Escherichia* | 1,2,3,5,6 | 38 | 6 |
| *Geobacter* | 2,6 | 1 | 5 |
| *Haemophilus* | 5 | 4 | n/a |
| *Helicobacter* | 4,5 | 2 | n/a |
| *Klebsiella* | 2 | 1 | 5 |
| *Legionella* | 2,4 | 25 | 5 |
| *Liberibacter* | 1 | 2 | 5 |
| *Mannheimia* | 1 | 1 | n/a |
| *Moraxella* | 5 | 5 | 3 |
| *Neisseria* | 1,5 | 3 | 5 |
| *Pseudomonas* | 1,2,3,5,6 | 50 | 3 |
| *Rhizobium* | 1,6 | 2 | 9 |
| *Salmonella* | 1,3,6 | 12 | 17 |
| *Serratia* | 1,5 | 4 | 5 |
| *Shigella* | 3,5 | 13 | 4 |
| *Sinorhizobium* | 1,3 | 2 | n/a |
| *Vibrio* | 1,2,6 | 15 | 9 |
| *Xanthomonas* | 2,3 | 12 | 8 |
| *Yersinia* | 3,5,6 | 9 | 5 |

**Table S3. Results of feature selection for each SVM model.** Features that were found commonly important across the three models are in bold.

| **Ranking** | **Features of three SVM Models** | | |
| --- | --- | --- | --- |
|  | ***N-terminal*** | ***C-terminal*** | ***Full*** |
| 1 | Cys | Glu | AvgCharge |
| 2 | **Asn** | **Asn** | Glu |
| 3 | **Ser** | **Ser** | Met |
| 4 | Thr | **Small** | **Asn** |
| 5 | Tiny | Non-Polar | Gln |
| 6 | **Small** | **Charged** | Arg |
| 7 | Aliphatic | Acidic | **Ser** |
| 8 | **Charged** | AF | Thr |
| 9 | Acidic | DG | Val |
| 10 | AE | EK | Tiny |
| 11 | CT | GK | **Small** |
| 12 | CV | GY | Aliphatic |
| 13 | EL | HF | Polar |
| 14 | EM | HL | **Charged** |
| 15 | GN | HN | CI |
| 16 | IE | HV | DS |
| 17 | IL | LW | DT |
| 18 | IW | MT | EC |
| 19 | MV | MW | EM |
| 20 | QT | RY | FC |
| 21 | SK | SA | GE |
| 22 | SS | ST | GN |
| 23 | TN | SW | HF |
| 24 | VW | TW | IE |
| 25 | YS | VL | ME |
| 26 | Helix | YS | MT |
| 27 | **Coil** | Beta Sheet | ND |
| 28 |  | **Coil** | NL |
| 29 |  |  | NN |
| 30 |  |  | NS |
| 31 |  |  | QR |
| 32 |  |  | QS |
| 33 |  |  | QT |
| 34 |  |  | RI |
| 35 |  |  | RV |
| 36 |  |  | SK |
| 37 |  |  | SN |
| 38 |  |  | SP |
| 39 |  |  | SR |
| 40 |  |  | SS |
| 41 |  |  | ST |
| 42 |  |  | SY |
| 43 |  |  | TN |
| 44 |  |  | TY |
| 45 |  |  | VE |
| 46 |  |  | VH |
| 47 |  |  | VV |
| 48 |  |  | WQ |
| 49 |  |  | YN |
| 50 |  |  | Helix |
| 51 |  |  | **Coil** |

**Table S4. Assessment of three integration schemes.** Shown are the accuracy values for each voting scheme. All 27 combinations of SVM models over three SVM kernels are explored.

| **Kernels** | | | **Integration type** | | |
| --- | --- | --- | --- | --- | --- |
| ***C-termini*** | ***N-termini*** | ***Full*** | ***Weak*** | ***Majority*** | ***Strict*** |
| Poly | Poly | Poly | 0.62 | 0.76 | 0.64 |
| Poly | Poly | RBF | 0.63 | 0.76 | 0.63 |
| Poly | Poly | Linear | 0.62 | 0.74 | 0.64 |
| Poly | RBF | Poly | 0.65 | 0.76 | 0.63 |
| Poly | RBF | RBF | 0.65 | 0.78 | 0.61 |
| Poly | RBF | Linear | 0.64 | 0.76 | 0.62 |
| Poly | Linear | Poly | 0.64 | 0.77 | 0.64 |
| Poly | Linear | RBF | 0.65 | 0.78 | 0.63 |
| Poly | Linear | Linear | 0.64 | 0.76 | 0.64 |
| RBF | Poly | Poly | 0.59 | 0.75 | 0.66 |
| RBF | Poly | RBF | 0.59 | 0.76 | 0.66 |
| RBF | Poly | Linear | 0.59 | 0.74 | 0.66 |
| RBF | RBF | Poly | 0.62 | 0.76 | 0.65 |
| RBF | RBF | RBF | 0.62 | 0.77 | 0.64 |
| RBF | RBF | Linear | 0.61 | 0.76 | 0.64 |
| RBF | Linear | Poly | 0.62 | 0.76 | 0.66 |
| RBF | Linear | RBF | 0.62 | 0.77 | 0.65 |
| RBF | Linear | Linear | 0.61 | 0.76 | 0.65 |
| Linear | Poly | Poly | 0.60 | 0.76 | 0.66 |
| Linear | Poly | RBF | 0.60 | 0.77 | 0.65 |
| Linear | Poly | Linear | 0.60 | 0.75 | 0.65 |
| Linear | RBF | Poly | 0.62 | 0.77 | 0.65 |
| Linear | RBF | RBF | 0.63 | 0.79 | 0.64 |
| Linear | RBF | Linear | 0.62 | 0.77 | 0.64 |
| Linear | Linear | Poly | 0.61 | 0.78 | 0.66 |
| Linear | Linear | RBF | 0.62 | 0.79 | 0.65 |
| Linear | Linear | Linear | 0.61 | 0.78 | 0.65 |

**Table S5. A detailed list of effectors in the positive set.** Each effector is included together with the species it belongs to and the secretion system it is identified with.

| **Id** | **Organism name** | **Secretion**  **system** | **UniProt ID** |
| --- | --- | --- | --- |
| 1 | Caulobacter crescentus | 1 | B8H2X0 |
| 2 | Liberibacter asiaticus | 1 | C6XHW4 |
| 3 | Liberibacter asiaticus | 1 | C6XI06 |
| 4 | Escherichia coli O44:H18 (strain 042 / EAEC) | 1 | D3H544 |
| 5 | Sinorhizobium meliloti | 1 | F7XG63 |
| 6 | Pseudomonas aeruginosa | 1 | O33415 |
| 7 | Escherichia coli | 1 | P01559 |
| 8 | Escherichia coli | 1 | P08715 |
| 9 | Escherichia coli | 1 | P22522 |
| 10 | Escherichia coli | 1 | P22542 |
| 11 | Pseudomonas aeruginosa | 1 | Q03023 |
| 12 | Erwinia chrysanthemi | 1 | Q07162 |
| 13 | Rhizobium leguminosarum bv. viciae (strain 3841) | 1 | Q1M7X8 |
| 14 | Ehrlichia chaffeensis | 1 | Q2GGE3 |
| 15 | Ehrlichia chaffeensis | 1 | Q2GHT8 |
| 16 | Ehrlichia chaffeensis | 1 | Q2GHU2 |
| 17 | Ehrlichia chaffeensis | 1 | Q2GI62 |
| 18 | Serratia marcescens | 1 | Q54450 |
| 19 | Serratia marcescens | 1 | Q59933 |
| 20 | Neisseria meningitidis | 1 | Q9K0K9 |
| 21 | Xanthomonas campestris pv. campestris (strain B100) | 2 | B0RNH9 |
| 22 | Xanthomonas campestris pv. campestris (strain B100) | 2 | B0RV82 |
| 23 | Burkholderia pseudomallei 1026b | 2 | I1WM41 |
| 24 | Burkholderia pseudomallei 1026b | 2 | I1WQ16 |
| 25 | Vibrio cholerae serotype O1 (strain ATCC 39315) | 2 | P01556 |
| 26 | Erwinia chrysanthemi | 2 | P04959 |
| 27 | Erwinia chrysanthemi | 2 | P07103 |
| 28 | Aeromonas hydrophila | 2 | P09167 |
| 29 | Erwinia chrysanthemi | 2 | P0C1A8 |
| 30 | Vibrio cholerae serotype O1 (strain ATCC 39315) | 2 | P0C6E9 |
| 31 | Aeromonas hydrophila | 2 | P10480 |
| 32 | Pseudomonas aeruginosa (strain ATCC 15692) | 2 | P14789 |
| 33 | Pseudomonas aeruginosa (strain ATCC 1569) | 2 | P26876 |
| 34 | Burkholderia cenocepacia (strain AU 1054) | 2 | Q1BN95 |
| 35 | Burkholderia cenocepacia (strain AU 1054) | 2 | Q1BW65 |
| 36 | Erwinia chrysanthemi | 2 | Q47499 |
| 37 | Aeromonas hydrophila | 2 | Q49KA8 |
| 38 | Legionella pneumophila (strain Paris) | 2 | Q5X2U6 |
| 39 | Legionella pneumophila (strain Paris) | 2 | Q5X611 |
| 40 | Legionella pneumophila (strain Paris) | 2 | Q5X650 |
| 41 | Legionella pneumophila (strain Paris) | 2 | Q5X7S3 |
| 42 | Legionella pneumophila (strain Paris) | 2 | Q5X960 |
| 43 | Erwinia carotovora subsp. atroseptica (strain SCRI 1043 / ATCC BAA-672) | 2 | Q6D5R0 |
| 44 | Erwinia carotovora subsp. atroseptica (strain SCRI 1043 / ATCC BAA-672) | 2 | Q6D8P1 |
| 45 | Acinetobacter sp. (strain ADP1) | 2 | Q6FD43 |
| 46 | Acinetobacter sp. (strain ADP1) | 2 | Q6FD56 |
| 47 | Vibrio vulnificus (strain YJ016) | 2 | Q7MDS1 |
| 48 | Vibrio vulnificus (strain YJ016) | 2 | Q7MID9 |
| 49 | Erwinia chrysanthemi | 2 | Q9APJ5 |
| 50 | Pseudomonas aeruginosa (strain ATCC 15692) | 2 | Q9I589 |
| 51 | Escherichia coli O127:H6 (strain E2348/69 / EPEC) | 3 | B7UMA0 |
| 52 | Erwinia amylovora (strain ATCC 49946) | 3 | D4IAU0 |
| 53 | Erwinia amylovora (strain ATCC 49946) | 3 | D4IAU5 |
| 54 | Salmonella typhimurium | 3 | O30916 |
| 55 | Pseudomonas aeruginosa | 3 | O34208 |
| 56 | Chlamydia trachomatis | 3 | O84091 |
| 57 | Chlamydia trachomatis | 3 | O84462 |
| 58 | Chlamydia trachomatis | 3 | P0CI27 |
| 59 | Salmonella typhimurium | 3 | P0CL52 |
| 60 | Escherichia coli | 3 | P0DJ90 |
| 61 | Yersinia enterocolitica | 3 | P15273 |
| 62 | Shigella Flexneri | 3 | P18012 |
| 63 | Shigella Flexneri | 3 | P33546 |
| 64 | Xanthomonas euvesicatoria | 3 | Q3BTM6 |
| 65 | Edwardsiella piscicida | 3 | Q4G4D4 |
| 66 | Pseudomonas syringae pv. syringae (strain B728a) | 3 | Q4ZLM6 |
| 67 | Pseudomonas syringae pv. syringae (strain B728a) | 3 | Q4ZX50 |
| 68 | Pseudomonas syringae pv. syringae (strain B728a) | 3 | Q4ZX80 |
| 69 | Pseudomonas aeruginosa | 3 | Q51445 |
| 70 | Pseudomonas syringae pv. tomato | 3 | Q52473 |
| 71 | Escherichia coli | 3 | Q5K5M1 |
| 72 | Burkholderia pseudomallei (strain K96243) | 3 | Q63K35 |
| 73 | Burkholderia pseudomallei (strain K96243) | 3 | Q63K50 |
| 74 | Xanthomonas euvesicatoria | 3 | Q6TQF0 |
| 75 | Shigella dysenteriae | 3 | Q7BQ98 |
| 76 | Shigella Flexneri | 3 | Q7BU69 |
| 77 | Pseudomonas syringae | 3 | Q7PC44 |
| 78 | Pseudomonas syringae pv. tomato | 3 | Q887D0 |
| 79 | Shigella Flexneri | 3 | Q8VSP9 |
| 80 | Yersinia enterocolitica | 3 | Q93KU8 |
| 81 | Shigella dysenteriae | 3 | Q9FBI2 |
| 82 | Coxiella burnetii (strain CbuG_Q212) | 4 | B6IYX2 |
| 83 | Coxiella burnetii (strain CbuG_Q212) | 4 | B6J209 |
| 84 | Coxiella burnetii (strain CbuG_Q212) | 4 | B6J3S1 |
| 85 | Bartonella henselae | 4 | I3QKD5 |
| 86 | Bartonella henselae | 4 | I3QKE3 |
| 87 | Bartonella henselae | 4 | I3QKE4 |
| 88 | Legionella pneumophila subsp. Pneumophila | 4 | I7I1E2 |
| 89 | Legionella pneumophila subsp. Pneumophila | 4 | I7I3V4 |
| 90 | Bordetella pertussis (strain Tohama) | 4 | P04977 |
| 91 | Bordetella pertussis (strain Tohama) | 4 | P04979 |
| 92 | Agrobacterium tumefaciens | 4 | P06668 |
| 93 | Bordetella pertussis (strain Tohama) | 4 | P0A3R5 |
| 94 | Agrobacterium tumefaciens | 4 | P0A3W8 |
| 95 | Helicobacter pylori | 4 | P55980 |
| 96 | Ehrlichia chaffeensis | 4 | Q2GG12 |
| 97 | Agrobacterium tumefaciens | 4 | Q44445 |
| 98 | Anaplasma marginale | 4 | Q5PAK8 |
| 99 | Anaplasma marginale | 4 | Q5PB23 |
| 100 | Anaplasma marginale | 4 | Q5PBL4 |
| 101 | Legionella pneumophila (strain Paris) | 4 | Q5X214 |
| 102 | Legionella pneumophila (strain Paris) | 4 | Q5X3V2 |
| 103 | Legionella pneumophila (strain Paris) | 4 | Q5X443 |
| 104 | Legionella pneumophila (strain Paris) | 4 | Q5X6G3 |
| 105 | Legionella pneumophila (strain Paris) | 4 | Q5X7D0 |
| 106 | Legionella pneumophila subsp. Pneumophila | 4 | Q5ZSM7 |
| 107 | Legionella pneumophila subsp. Pneumophila | 4 | Q5ZSQ2 |
| 108 | Legionella pneumophila subsp. Pneumophila | 4 | Q5ZV00 |
| 109 | Legionella pneumophila subsp. Pneumophila | 4 | Q5ZYI2 |
| 110 | Agrobacterium tumefaciens | 4 | Q79AT3 |
| 111 | Moraxella catarrhalis | 4 | Q9R6B6 |
| 112 | Pseudomonas aeruginosa | 5 | A1YKX3 |
| 113 | Escherichia coli O44:H18 (strain 042 / EAEC) | 5 | O33407 |
| 114 | Serratia marcescens | 5 | O68900 |
| 115 | Yersinia enterocolitica | 5 | P09489 |
| 116 | Bordetella pertussis (strain Tohama) | 5 | P0C2W0 |
| 117 | Serratia marcescens | 5 | P14283 |
| 118 | Dichelobacter nodosus | 5 | P15320 |
| 119 | Haemophilus influenzae | 5 | P42779 |
| 120 | Helicobactor pylori | 5 | P45387 |
| 121 | Escherichia coli | 5 | P55981 |
| 122 | Moraxella catarrhalis | 5 | Q03155 |
| 123 | Bordetella pertussis (strain Tohama) | 5 | Q08657 |
| 124 | Escherichia coli | 5 | Q45340 |
| 125 | Haemophilus influenzae | 5 | Q47692 |
| 126 | Haemophilus influenzae | 5 | Q48031 |
| 127 | Aggregatibacter actinomycetemcomitans | 5 | Q48152 |
| 128 | Moraxella catarrhalis | 5 | Q6VBQ2 |
| 129 | Bordetella pertussis (strain Tohama) | 5 | Q79GN7 |
| 130 | Shigella Flexneri | 5 | Q7BCK4 |
| 131 | Bordetella pertussis (strain Tohama) | 5 | Q7VZ27 |
| 132 | Neisseria meningitidis serogroup B (strain MC58) | 5 | Q8GH87 |
| 133 | Moraxella catarrhalis | 5 | Q8KQM9 |
| 134 | Pseudomonas aeruginosa | 5 | Q9HVN7 |
| 135 | Pseudomonas aeruginosa | 5 | Q9HWU6 |
| 136 | Neisseria meningitidis | 5 | Q9K0T0 |
| 137 | Moraxella catarrhalis | 5 | Q9L961 |
| 138 | Escherichia coli O78:H11 (strain H10407 / ETEC) | 5 | Q9XD84 |
| 139 | Yersinia pseudotuberculosis | 6 | A0A0H3B7J5 |
| 140 | Burkholderia mallei | 6 | A5PM30 |
| 141 | Edwardsiella piscicida | 6 | A8YQR4 |
| 142 | Agrobacterium tumefaciens | 6 | A9CFH2 |
| 143 | Agrobacterium tumefaciens C58 | 6 | A9CGH1 |
| 144 | Burkholderia cenocepacia | 6 | B4EMB9 |
| 145 | Salmonella gallinarum (strain 287/91) | 6 | B5R7C3 |
| 146 | Escherichia coli | 6 | D3GUW5 |
| 147 | Dickeya dadantii | 6 | E0SAK8 |
| 148 | Shiga toxin-producing E.coli | 6 | P46855 |
| 149 | Rhizobium leguminosarum bv. viciae (strain 3841) | 6 | Q1MFR3 |
| 150 | Burkholderia thailendensis | 6 | Q2T2K7 |
| 151 | Burkholderia thailendensis | 6 | Q2T422 |
| 152 | Burkholderia thailendensis | 6 | Q2T6Y9 |
| 153 | Pseudomonas fluorescens | 6 | Q4K5B7 |
| 154 | Pseudomonas protegens | 6 | Q4KC90 |
| 155 | Enterohemorragic E. coli | 6 | Q8XDQ1 |
| 156 | Salmonella typhimurium | 6 | Q93IS4 |
| 157 | Pseudomonas aeruginosa | 6 | Q9HU94 |
| 158 | Pseudomonas aeruginosa | 6 | Q9HYC2 |
| 159 | Pseudomonas aeruginosa | 6 | Q9HYC5 |
| 160 | Pseudomonas aeruginosa | 6 | Q9I0E0 |
| 161 | Pseudomonas aeruginosa | 6 | Q9I0F4 |
| 162 | Pseudomonas aeruginosa | 6 | Q9I2Q1 |
| 163 | Pseudomonas aeruginosa | 6 | Q9I3K2 |
| 164 | Pseudomonas aeruginosa | 6 | Q9I739 |
| 165 | Vibrio cholerae | 6 | Q9KMN9 |
| 166 | Vibrio cholerae serotype O1 (strain ATCC 39315) | 6 | Q9KNE5 |
| 167 | Vibrio cholerae serotype O1 (strain ATCC 39315) | 6 | Q9KS43 |
| 168 | Vibrio cholerae | 6 | Q9KS45 |

**Table S6. GO annotation terms used in functional enrichment analysis.** Shown is the list of second-level terms used in the analysis.

| ***GO TERMS*** | ***Function*** | ***High-level class*** |
| --- | --- | --- |
| GO:0000003 | reproduction | Biological_process |
| GO:0000988 | protein binding transcription factor activity | Molecular_Function |
| GO:0001071 | nucleic acid binding transcription factor activity | Molecular_Function |
| GO:0001906 | cell killing | Biological_process |
| GO:0002376 | immune system process | Biological_process |
| GO:0003824 | catalytic activity | Molecular_Function |
| GO:0004872 | receptor activity | Molecular_Function |
| GO:0005198 | structural molecule activity | Molecular_Function |
| GO:0005215 | transporter activity | Molecular_Function |
| GO:0005488 | binding | Molecular_Function |
| GO:0005576 | extracellular region | Cellular_component |
| GO:0005623 | cell | Cellular_component |
| GO:0008152 | metabolic process | Biological_process |
| GO:0009055 | electron carrier activity | Molecular_Function |
| GO:0009295 | nucleoid | Cellular_component |
| GO:0009987 | cellular process | Biological_process |
| GO:0016015 | morphogen activity | Molecular_Function |
| GO:0016020 | membrane | Cellular_component |
| GO:0016209 | antioxidant activity | Molecular_Function |
| GO:0016247 | channel regulator activity | Molecular_Function |
| GO:0016530 | metallochaperone activity | Molecular_Function |
| GO:0019012 | virion | Cellular_component |
| GO:0022414 | reproductive process | Biological_process |
| GO:0022610 | biological adhesion | Biological_process |
| GO:0023052 | signaling | Biological_process |
| GO:0030054 | cell junction | Cellular_component |
| GO:0030234 | enzyme regulator activity | Molecular_Function |
| GO:0030545 | receptor regulator activity | Molecular_Function |
| GO:0031012 | extracellular matrix | Cellular_component |
| GO:0031386 | protein tag | Molecular_Function |
| GO:0031974 | membrane-enclosed lumen | Cellular_component |
| GO:0032501 | multicellular organismal process | Biological_process |
| GO:0032502 | developmental process | Biological_process |
| GO:0032991 | macromolecular complex | Cellular_component |
| GO:0036370 | D-alanyl carrier activity | Molecular_Function |
| GO:0040007 | growth | Biological_process |
| GO:0040011 | locomotion | Biological_process |
| GO:0042056 | chemoattractant activity | Molecular_Function |
| GO:0043226 | organelle | Cellular_component |
| GO:0044420 | extracellular matrix component | Cellular_component |
| GO:0044421 | extracellular region part | Cellular_component |
| GO:0044422 | organelle part | Cellular_component |
| GO:0044423 | virion part | Cellular_component |
| GO:0044425 | membrane part | Cellular_component |
| GO:0044456 | synapse part | Cellular_component |
| GO:0044464 | cell part | Cellular_component |
| GO:0044699 | single-organism process | Biological_process |
| GO:0045182 | translation regulator activity | Molecular_Function |
| GO:0045202 | synapse | Cellular_component |
| GO:0045499 | chemorepellent activity | Molecular_Function |
| GO:0045735 | nutrient reservoir activity | Molecular_Function |
| GO:0048511 | rhythmic process | Biological_process |
| GO:0050896 | response to stimulus | Biological_process |
| GO:0051179 | localization | Biological_process |
| GO:0051234 | establishment of localization | Biological_process |
| GO:0051704 | multi-organism process | Biological_process |
| GO:0055044 | symplast | Cellular_component |
| GO:0060089 | molecular transducer activity | Molecular_Function |
| GO:0065007 | biological regulation | Biological_process |
| GO:0097423 | mitochondrion-associated adherens complex | Cellular_component |

**Table S7. Leave-one-out (LOO), 10–fold cross validation (CV), and test set assessments for N-terminal SVM and Random Forest (RF) models.** For each assessment type, the performance using one of the three different kernels is measured by Accuracy (Acc), Precision (Pre), Recall (Rec), and f-measure. The default probability threshold of $\theta_{0}=0.5$ is used.

| **Evaluation** | **Kernel** | **Acc** | **Pre** | **Rec** | **f-measure** |
| --- | --- | --- | --- | --- | --- |
| 10-fold CV | Radial | 0.58 | 0.57 | 0.66 | 0.61 |
|  | Linear | 0.64 | 0.64 | 0.67 | 0.65 |
|  | Polynomial | 0.61 | 0.59 | 0.74 | 0.65 |
|  | RF | 0.62 | 0.62 | 0.62 | 0.62 |
| LOO | Radial | 0.80 | 0.80 | 0.80 | 0.80 |
|  | Linear | 0.76 | 0.77 | 0.76 | 0.76 |
|  | Polynomial | 0.68 | 0.73 | 0.68 | 0.67 |
|  | RF | 0.71 | 0.71 | 0.71 | 0.71 |

**Table S8. Leave-one-out (LOO), 10–fold cross validation (CV), and test set assessments for C-terminal SVM and Random Forest (RF) models.** For each assessment type, the performance using one of the three different kernels is measured by Accuracy (Acc), Precision (Pre), Recall (Rec), and f-measure. The default probability threshold of $\theta_{0}=0.5$ is used.

| **Evaluation** | **Kernel** | **Acc** | **Pre** | **Rec** | **f-measure** |
| --- | --- | --- | --- | --- | --- |
| 10-fold CV | Radial | 0.56 | 0.56 | 0.56 | 0.56 |
|  | Linear | 0.57 | 0.58 | 0.55 | 0.56 |
|  | Polynomial | 0.56 | 0.61 | 0.42 | 0.49 |
|  | RF | 0.62 | 0.62 | 0.62 | 0.61 |
| LOO | Radial | 0.73 | 0.73 | 0.73 | 0.73 |
|  | Linear | 0.70 | 0.70 | 0.70 | 0.70 |
|  | Polynomial | 0.70 | 0.70 | 0.70 | 0.70 |
|  | RF | 0.68 | 0.68 | 0.68 | 0.68 |

**Table S9. Leave-one-out (LOO), 10–fold cross validation (CV), and test set assessments for the full sequence SVM and Random Forest (RF) models.** For each assessment type, the performance using one of the three different kernels is measured by Accuracy (Acc), Precision (Pre), Recall (Rec), and f-measure. The default probability threshold of $\theta_{0}=0.5$ is used.

| **Evaluation** | **Kernel** | **Acc** | **Pre** | **Rec** | **f-measure** |
| --- | --- | --- | --- | --- | --- |
| 10-fold CV | Radial | 0.85 | 0.88 | 0.82 | 0.84 |
|  | Linear | 0.83 | 0.84 | 0.81 | 0.82 |
|  | Polynomial | 0.84 | 0.90 | 0.78 | 0.83 |
|  | RF | 0.87 | 0.87 | 0.87 | 0.87 |
| LOO | Radial | 0.87 | 0.87 | 0.87 | 0.87 |
|  | Linear | 0.83 | 0.83 | 0.83 | 0.83 |
|  | Polynomial | 0.81 | 0.84 | 0.81 | 0.81 |
|  | RF | 0.89 | 0.89 | 0.89 | 0.89 |

**Table S10. Grid search of the probability threshold** $\theta_{0}$ **for a full SVM model with polynomial kernel and 10-fold cross validation assessment.** While the original prediction probability uses the probability threshold $\theta_{0}=0.5$, the optimal, with respect to the accuracy probability threshold of $\theta_{0}=0.4$ increases the false positive ratio and is not considered. In turn, the most stringent threshold of $\theta_{0}=0.9$ drastically decreases the false positive rate, while significantly decreasing accuracy by 0.16. Abbreviations: TP – true positives, TN – true negatives, FP – false positives, FN – false negatives, FPR – false positive rate, TPR – true positive rate.

| ***θ*** | ***TP*** | ***TN*** | ***FP*** | ***FN*** | ***FPR*** | ***TPR*** | ***Accuracy*** |
| --- | --- | --- | --- | --- | --- | --- | --- |
| 0.1 | 15.8 | 6.6 | 10.4 | 1.2 | 0.61 | 0.93 | 0.66 |
| 0.2 | 15.4 | 9.2 | 7.8 | 1.6 | 0.46 | 0.91 | 0.72 |
| 0.3 | 15.1 | 11.9 | 5.1 | 1.9 | 0.30 | 0.89 | 0.79 |
| 0.4 | 15.1 | 14.2 | 2.8 | 1.9 | 0.16 | 0.89 | 0.86 |
| 0.5 | 13.2 | 15.4 | 1.6 | 3.8 | 0.09 | 0.78 | 0.84 |
| 0.6 | 11.0 | 15.6 | 1.4 | 6.0 | 0.08 | 0.65 | 0.78 |
| 0.7 | 10.0 | 16.2 | 0.8 | 7.0 | 0.05 | 0.59 | 0.77 |
| 0.8 | 8.6 | 16.5 | 0.5 | 8.4 | 0.03 | 0.51 | 0.74 |
| 0.9 | 6.7 | 16.5 | 0.5 | 10.3 | 0.03 | 0.39 | 0.68 |

**Table S11. Comparative performance analysis of state-of-the-art effector candidate prediction methods on our dataset.** The application of the methods that are designed to detect effector candidates of a single secretion system on the dataset of 168 effectors and 168 non-effectors has showed that none of them can be used universally across all six secretion systems.

| **Method** | **BPBAac** | **T3_MM** | **T4SEpre_bpbAac** | **T4SEpre_psAac** | **EffectiveT3** | **Sieve** |
| --- | --- | --- | --- | --- | --- | --- |
| Accuracy | 0.45 | 0.66 | 0.58 | 0.58 | 0.58 | 0.52 |
| Recall | 0.13 | 0.48 | 0.18 | 0.16 | 0.29 | 0.04 |

**Table S12. Recall values across six secretion systems for each state-of-the-art method applied to our dataset.** One asterisk corresponds to the methods specialized in predicting T3SS effector candidates, while two asterisks correspond to the methods specialized in predicting T4SS effector candidates.

| **Secretion**  **System** | **PREFFECTOR** | **BPBAac*** | **T3_MM*** | **T4SEpre_bpbAac**** | **T4SEpre_psAac**** | **EffectiveT3*/**** | **Sieve**  ***** |
| --- | --- | --- | --- | --- | --- | --- | --- |
| T1SS | 0.85 | 0.09 | 0.43 | 0.00 | 0.00 | 0.27 | 0.00 |
| T2SS | 0.97 | 0.00 | 0.33 | 0.00 | 0.03 | 0.00 | 0.00 |
| T3SS* | 0.84 | 0.61 | 0.84 | 0.10 | 0.06 | 0.71 | 0.23 |
| T4SS** | 0.77 | 0.07 | 0.47 | 0.77 | 0.77 | 0.27 | 0.00 |
| T5SS | 1.00 | 0.00 | 0.50 | 0.07 | 0.00 | 0.14 | 0.00 |
| T6SS | 0.87 | 0.00 | 0.27 | 0.10 | 0.03 | 0.30 | 0.00 |

**Table S13. Whole-genome application of PREFFECTOR to four Gram-negative bacterial species.** Shown are application results for the default optimal (Random Forest) and SVM with extremely stringent threshold (*θ*=0.9) predictions. The smaller number of known effectors for the classifier that uses the extremely stringent threshold is due to a higher number of false negatives.

| **Species** | **Disease** | **Genome size, Mb** | **N of  genes** | **Known effectors** | **Predicted using**  ***RF*** | | **Predicted using SVM, *θ*=0.9** | |
| --- | --- | --- | --- | --- | --- | --- | --- | --- |
|  |  |  |  |  | **Total** | **N of known** | **Total** | **N of known** |
| *Acinetobacter baumanni* | Meningitis | 3.98 | 3,715 | 5 | 753 | 5 | 260 | 4 |
| *Chlamydia trachoma* | Chlamydia infection, trachoma | 1.04 | 926 | 30 | 204 | 18 | 74 | 8 |
| *Helicobacter pylori* | Gastritis | 1.67 | 1,485 | 3 | 457 | 3 | 119 | 2 |
| *Legionella pneumophila* | Legionellosis | 3.40 | 3,025 | 134 | 989 | 105 | 238 | 42 |
| *Salmonella enterica* | Salmonellosis | 4.58 | 4,547 | 43 | 708 | 31 | 194 | 8 |
| *Yersinia pseudotuberculosis* | Far East scarlet-like fever | 4.72 | 4,164 | 6 | 886 | 6 | 272 | 2 |

**Table S14. Whole-genome application of specialized state-of-the-art prediction methods to the five Gram-negative bacterial species considered in the paper.** Shown are the numbers of genes predicted as effector candidates. Only stand-alone software packages were used in this application.

| ***Method  (secretion system)*** | ***Chlamydia (III)*** | ***Helicobacter (IV, V)*** | ***Legionella (I, II, IV, V)*** | ***Salmonella (III)*** | ***Yersenia (III*)** |
| --- | --- | --- | --- | --- | --- |
| BPBAac (III) | 39 | 9 | 38 | 50 | 55 |
| T3_MM (III) | 204 | 393 | 609 | 517 | 713 |
| T4SEpre_bpbAac (IV) | 38 | 84 | 320 | 74 | 83 |
| T4SEpre_psAac (IV) | 20 | 38 | 258 | 24 | 28 |
